# Identification of the molecular characteristics of *Bacillus anthracis* (1982-2020) isolates in East Indonesia using multilocus variable-number tandem repeat analysis

**DOI:** 10.14202/vetworld.2022.953-961

**Published:** 2022-04-16

**Authors:** D. W. Yudianingtyas, B. Sumiarto, H. Susetya, Mo Salman, T. F. Djatmikowati, Haeriah Haeriah, Abdul Rahman, R. Mangidi

**Affiliations:** 1 Doctoral Study Program, Faculty of Veterinary Medicine, Universitas Gadjah Mada, Yogyakarta, Indonesia; 2 Department of Epidemiology and Veterinary Information, Disease Investigation Centre Maros, Directorate General of Livestock Services and Animal Health, Ministry of Agriculture, The Republic of Indonesia, Indonesia; 3 Department of Veterinary Public Health, Faculty of Veterinary Medicine, Universitas Gadjah Mada, Yogyakarta, Indonesia; 4 Department of Clinical Sciences, Animal Population Health Institute, College of Veterinary Medicine and Biomedical Science, Colorado State University, Fort Collins, United States of America; 5 Bacteriology laboratory, Disease Investigation Centre Maros, Directorate General of Livestock Services and Animal Health, Ministry of Agriculture, Indonesia, The Republic of Indonesia, Indonesia; 6 Disease Investigation Centre Maros, Directorate General of Livestock Services and Animal Health, Ministry of Agriculture, The Republic of Indonesia, Indonesia

**Keywords:** Bacillus anthracis, Indonesia, multilocus variable-number tandem-repeat analysis-8

## Abstract

**Background and Aim:**

Anthrax is one of the endemic strategic diseases in East Indonesia, particularly in the provinces of South Sulawesi, West Sulawesi, Gorontalo, East Nusa Tenggara, and West Nusa Tenggara. Anthrax is an important disease due to its zoonotic and economic impact on the livestock industry. This study aimed to identify the molecular characteristics of *Bacillus anthracis* in East Indonesia using multilocus variable-number tandem repeat (VNTR) analysis (MLVA).

**Materials and Methods:**

Isolates were obtained from an investigation of anthrax outbreaks in five provinces of East Indonesia from 1982 to 2020. Conventional polymerase chain reaction for *B. anthracis* was used to identify MLVA-8. Deoxyribonucleic acid sequencing analysis was based on MLVA-8 primers for VNTR identification of the phylogenetic relationship among 24 isolates of *B. anthracis* obtained from 17 distinct districts/cities in East Indonesia. Tandem Repeats Finder was used for VNTR identification, and Molecular Evolutionary Genetics Analysis X was used to construct phylogenetic analysis.

**Results:**

In this study, 24 isolates were classified as genotype or lineage A. There were four subgroups of *B. anthracis* circulating in East Indonesia based on eight molecular marker loci sequence results.

**Conclusion:**

The findings of this study show that MLVA-8 typing might be useful as a subtyping tool for the epidemiological investigation of identical genotypes and low genetic diversity of *B. anthracis*. No other lineage of *B. anthracis* was circulating in East Indonesia. Other molecular methods are needed, such as extended MLVA, whole-genome sequencing, and canonical single-nucleotide polymorphism, for a more precise study of *B. anthracis* genetic diversity.

## Introduction

Anthrax is caused by *Bacillus anthracis*, a rod-shaped, encapsulated, and spore-forming bacterium [1-4]. It is one of the important and strategic diseases in Indonesia, which is concerning for public health authorities [5]. Anthrax in Indonesia was introduced in 1885 (i.e. during the colonial period) and has spread to the entire country. At present, 14 provinces in Indonesia experience anthrax endemically. These provinces include South Sulawesi, West Sulawesi, Gorontalo, East Nusa Tenggara, and West Nusa Tenggara, located in the eastern part of Indonesia [5]. Anthrax became one of the major infectious diseases among animals in East Indonesia, particularly in the South Sulawesi province, which is the source of livestock for other provinces, including Sulawesi Island, Maluku North, Maluku, Papua, and West Papua.

A genetic diversity study of *B. anthracis* has been performed worldwide using several techniques, such as amplified fragment length polymorphism [6], multilocus variable-number tandem repeat (VNTR) analysis (MLVA) [7-22], single-nucleotide polymorphism (SNP) [1,2,6,7,12,14-19,23-27], and whole-genome sequencing (WGS) [1,2,6,7,22,23,28]. MLVA detects VNTRs through polymerase chain reaction (PCR) based on target genes, which usually contain low-frequency mutations in the bacterial genome [29-31]. A genetic diversity study of *B. anthracis* using MLVA is the basic study for bacterial molecular genotyping [10,11,23,32], but it has never been conducted in Indonesia.

Furthermore, the phylogenetic diversity of *B. anthracis* in East Indonesia has not been investigated. *B. anthracis* circulating worldwide has been classified into lineages A, B, and C. Most isolates in Asia, including Indonesia, are within lineage A [7-10]. A previous study was to determine the link between two *B. anthracis* isolates from South Sulawesi (Pangkajene Kepulauan) and Bekasi (West Java) classified isolates as A3.a and A3.b genotypes based on the MLVA technique [7].

This study aimed to identify the molecular characteristic of *B. anthracis* in East Indonesia using MLVA-8 method, as part of a retrospective investigation of anthrax outbreaks from 1982 to 2020.

## Materials and Methods

### Ethical approval

The study was approved by Medical Health Research Ethics Committee, Faculty of Medicine, Universitas Gadjah Mada, with approval no. KE/FK0428/EC/2019*. B. anthracis* isolates used in this study were collected by the Disease Investigation Center Maros (DIC Maros), which is authorized as an anthrax reference and veterinary diagnostic laboratory mandated by the Ministry of Agriculture of Indonesia. Diagnosis confirmation, outbreak investigation, and animal health surveillance are part of DIC Maros roles and tasks.

### Study period and location

This study was conducted from August 2019 to March 2021, consisting of: 1) *B. anthracis* isolate restore and selection based on morphology colony observation; 2) data information collection of selected *B. anthracis* isolates, 3) molecular identification using multiplex conventional PCR; 4) MLVA-8 PCR, and 5) DNA sequencing. Steps 1-4 were conducted at DIC Maros (Bacteriology Laboratory; Epidemiology and Veterinary Information Department; and Biotechnology Laboratory). The last step, DNA sequencing, was conducted at 1**^st^** BASE, Malaysia.

### Data information and geographic distribution of *B. anthracis* isolates

**Figure-1:**
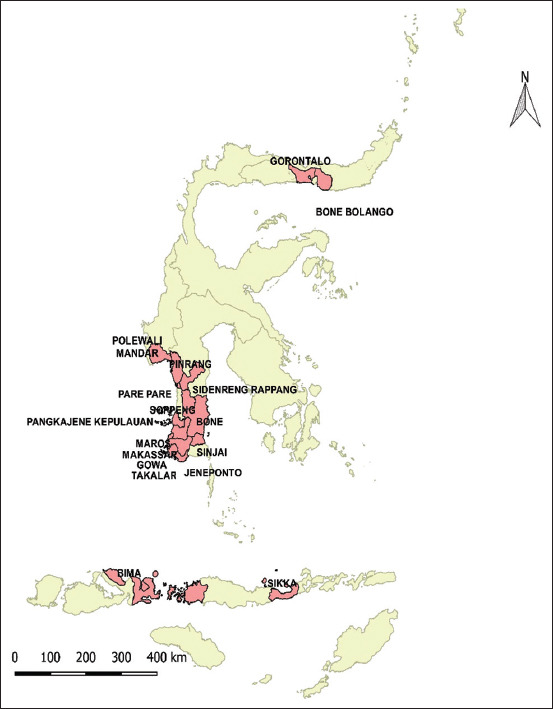
Geographical distribution of Bacillus anthracis isolates used in this study [Source: Map generated using QGIS software].

*B. anthracis* isolates were selected based on their geographical source by district/city (Figure-1) as well as four old isolates (1982-2004) from the available archival collection from DIC Maros (Table-1). The newest isolate was collected from the recent 2020 anthrax outbreak in Soppeng district. Most isolates were related to livestock anthrax cases, except one isolate, which was obtained from deer (isolate number 10, Table-1).

**Table 1:** Data of 24 *Bacillus anthracis* isolates used in this study.

S. No.	Isolates number	Year collected	Province	District	Species infected	Origin of sample
1.	07820944	1982	South Sulawesi	Bone	Bali cattle	Blood
2.	07840716	1984	South Sulawesi	Pangkajene Kepulauan	Bali cattle	Blood
3.	07850053	1985	South Sulawesi	Maros	Bali cattle	Blood/Ear
4.	07040472	2004	East Nusa Tenggara	Sikka	Bali cattle	Contaminated soil
5.	07150743	2015	South Sulawesi	Gowa	Bali cattle	Contaminated soil
6.	07150615	2015	South Sulawesi	Maros	Bali cattle	Ear
7.	07151054	2015	South Sulawesi	Parepare	Bali cattle	Contaminated soil
8.	07160229	2016	West Sulawesi	Polewali Mandar	Goat	Blood/contaminated soil
9.	07160171	2016	South Sulawesi	Pinrang	Bali cattle	Blood/contaminated soil
10.	07160062	2016	South Sulawesi	Gowa	Deer	Organ (Lien, liver)
11.	07160128	2016	South Sulawesi	Makassar	Bali cattle	Blood/contaminated soil
12.	07160129	2016	West Nusa Tenggara	Bima	Bali cattle	Blood swab
13.	07170669	2016	Gorontalo	Bone Bolango	Bali cattle	Contaminated soil
14.	07170670	2017	Gorontalo	Bone Bolango	Bali cattle	Ear
15.	07170997	2017	Gorontalo	Gorontalo	Bali cattle	Lien
16.	07170703	2017	South Sulawesi	Sidenreng Rappang	Bali cattle	Contaminated soil
17.	A07180115	2018	South Sulawesi	Gowa	Horse	Contaminated soil
18.	A07190011	2019	South Sulawesi	Jeneponto	Horse	Contaminated soil
19.	A07180083	2019	South Sulawesi	Takalar	Horse	Contaminated soil
20.	A07190097	2019	South Sulawesi	Bone	Bali cattle	Contaminated soil
21.	A07190134	2019	South Sulawesi	Maros	Bali cattle	Contaminated soil
22.	A07190228	2019	South Sulawesi	Sinjai	Bali cattle	Lung
23.	P07190794	2019	East Nusa Tenggara	West Manggarai	Bali cattle	Contaminated soil
24.	A07200012	2020	South Sulawesi	Soppeng	Bali cattle	Contaminated soil

The area coverage of DIC Maros includes 10 provinces in Sulawesi Island, Maluku Island, North Maluku Island, and Papua Island. East Nusa Tenggara and West Nusa Tenggara provinces are under DIC Denpasar, located in the Bali province, which is categorized as an anthrax-free area. Therefore, for anthrax diagnostics, all samples from both provinces were sent and processed at DIC Maros.

Most isolates were derived from the anthrax-endemic areas, which have repeated or sporadic cases every year (DIC Maros data, unpublished). Of the 13 anthrax history districts/cities in South Sulawesi, 11 isolates from 11 districts/cities were successfully collected and cultivated for MLVA-8. Isolates from Barru district were not revived, and there were no archived isolates from two other districts, Bulukumba and North Luwu.

### Analysis of *B. anthracis* isolates

Twenty-four *B. anthracis* field isolates were collected from the DIC Maros areas during the outbreak investigations from 1982 to 2020. All the isolates were related to anthrax outbreak/cases. The original samples were blood, contaminated soil, and organs. Epidemiologic data for each isolate are provided in a section of DIC Maros (Table-1). All isolates were characterized morphologically during the diagnostic and storage process based on the World Organization for Animal Health anthrax diagnostic procedure and under safety procedure as DIC Maros holds International Organization for Standardization/International Electrotechnical Commission 17025:2017 [2]. Each isolate was inoculated into sheep blood agar and followed by macroscopic observation (flat or slightly convex, non-hemolytic, rough colony, irregular edges, ground-glass appearance, medusa head, and sticky consistency when manipulated with an inoculating loop).

### Deoxyribonucleic acid (DNA) isolation

The DNA of *B. anthracis* colonies was extracted using InstaGene Matrix (Bio-Rad Laboratories, Herculas, CA, USA), according to the manufacturer’s instructions. Pre-treatment heating was performed at 110°C for 15 min for colony bacterial suspension and bacteria inactivation (researcher’s modification). Each isolate suspension was reinoculated into sheep blood agar to investigate complete inactivation before the DNA extraction process.

### Multiplex PCR, VNTR PCR assay, and DNA sequencing

Conventional PCR was conducted for chromosomal and plasmid marker detection of *B. anthracis* following a previous study [33]. Three pairs of primers were used for the chromosomal gene detection (152 bp), the lethal factor virulence gene located on pXO1 (385 bp), and the capsule gene located on pXO2 (264 bp) [20]. Primer concentration was 18 pmoL. The three pairs of primers used were chromosomal detection (152 bp), 385 bp segment of the lethal factor virulence gene on pXO1, and a 264 bp segment of a capsule gene on pXO2 [20]. The Master Mix PCR was prepared inside a clean PCR suite. The PCR mixture (25 μL) contained 12.5 μL of HotStarTaq Master Mix, 1.25 μL of primer Ba813 R1, 1.25 μL of primer Ba813 R2, 1.25 μL of primer Cap 57, 1.25 μL of primer Cap 58, 1.25 μL of primer Lef 3, 1.25 μL of primer Lef 4, 2 μL of DNA template, and 3 μL of H_2_O. Amplification was performed using the Rotor-Gene Q PCR thermal cycler with the following PCR conditions: (1) Pre-denaturation at 95°C for 15 min (1 cycle); (2) denaturation at 94°C for 40 s, annealing at 54°C for 40 s (35 cycles), and extension at 68°C for 40 s (35 cycles); and then (3) final extension on 68°C for 7 min.

The MLVA-8 assay was performed using eight pairs of primers [7], consisting of six chromosomal (vrrA, vrrB1, vrrB2, vrrC1, vrrC2, and CG3) and two plasmid markers (pXO1-aat and pXO2-at). The primer concentration was 10 pmoL. Each VNTR marker was amplified separately using PCR, with the PCR mixture (25 μL) containing 12.5 μL of HotStarTaq Master mix, 1.5 μL of each forward and reverse primer, 5 μL of DNA template, and 4.5 μL of H_2_O. PCR was performed as per the above-mentioned PCR conditions. The DNA of PCR products was visualized using gel electrophoresis, separated in 3% agarose gel (UltraPure™ Agarose, Invitrogen).

### Setting of MLVA-8 assay

Two isolates, the vaccine strain (Sterne 34F, Anthravet^®^, Pusvetma, Indonesia) and one of the old isolates from Pangkajene Kepulauan (isolate no. 2, Table-1), were used as quality control for MLVA-8. The Sterne 43F (Anthravet^®^, Pusvetma) vaccine strain had a complete genome sequence in GenBank, and an isolate from Pangkajene Kepulauan was classified as A3 lineage from previously published articles [7]. Both isolates were tested to be duplicates during the whole laboratory process, from bacterial reculture until the DNA sequencing process.

### DNA sequencing

To ensure that the calculation of the number of repeats was correct, each amplicon (PCR products) representing all the different alleles of each VNTR locus was sequenced. For sequencing, the same primers as those for amplification were used. All PCR products were sequenced at the 1^st^ BASE, Malaysia, using the Sanger sequencing method. The amplicon size of MLVA-8 markers was defined by DNA sequence results and compared to reference genotypes [7-9].

### VNTR identification and construction of the phylogenetic tree

Tandem Repeats Finder (TRF) version 4.09 (https://tandem.bu.edu/trf/trf.html) was used to determine the number of tandem repeats in each amplicon [34]. The discriminatory power of each VNTR marker was calculated according to Simpson’s formula [35,36] using an online program (http://insilico.ehu.es/mini_tools/discriminatory_power/index.php).

Among 36 field isolates, 24 isolates and vaccine strains were selected. Only the isolates that showed well-defined PCR DNA sequencing results were processed for dendrogram construction. Phylogenetic analysis was used to distinguish the relationship between *B. anthracis* isolates based on the variation in the VNTRs, which was analyzed using the neighbor-joining p-distance method [37-40]. A phylogenetic tree was constructed using the Molecular Evolutionary Genetics Analysis (MEGA) X [37] software (https://www.megasoftware.net). Data from forward and reverse sequencing primers were combined and aligned manually. Four reference strains (CDC 684, London 499, JF 3960, and JF 3965) from GenBank for VNTR were included for identification, and the phylogenetic tree construction was based on the Basic Local Alignment Search Tool results.

## Results

### Conventional PCR identification

**Figure-2:**
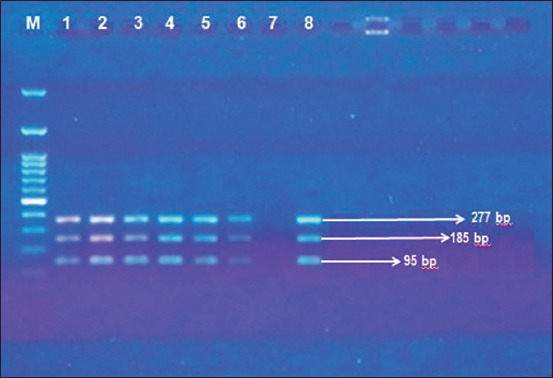
Multiplex polymerase chain reaction for Bacillus anthracis identification using Ramisse’s [33] protocol, Ba813 (chromosome): 277 bp, Lef: 185 bp, and Cap: 95 bp; lane 1: Marker 50 bp, lanes 2-4, 5, 6, and 8: Samples/field isolates, and lane 7: Non-template control.

*B. anthrax* identification using conventional PCR was performed with a selection process before MLVA-8. All the isolates selected should have amplicons for three gene targets (chromosomal, lef, and cap) to be able to obtain better information from eight marker loci results. Gene targets amplicons of multiplex PCR were visualized as shown in Figure-2 [33]. 

### Profile of MLVA-8 and VNTR identification

The sample was processed based on the identification of *B. anthracis* by PCR and the MLVA-8 assay [7]. Table-2 presents the VNTR identification and calculation using the TRF program for each chromosomal and plasmid marker. Diversity among 24 *B. anthracis* isolates was observed based on each VNTR fragment size, tandem repeat, and discriminatory power. The evolutionary pattern of nucleotide differences among each VNTR locus of 24 *B. anthracis* isolates exhibited allelic variations, frequency of allele, and percentage of insertions and deletions [34].

**Table 2:** MLVA-8 profile of 24 field isolates and vaccine.

S. No.	ID	Amplicon size^a^															

		vrrA	No. repeat	vrrB1	No. repeat	vrrB2	No. repeat	vrrC1	No. repeat	vrrC2	No. repeat	CG3	No. repeat	pXO1	No. repeat	pXO2	No. repeat
1.	07820944	317	5.4	233	15.4	163	3.7	620	10.3	536	8.6	160	2.5	129	7.3	147	10.5
2.	07840716	317	5.4	232	15.4	167	3.7	620	10.3	536	8.6	160	2.4	139	9	147	10.5
3.	07850053	317	5.4	237	6.9	163	3.7	621	10.3	537	8.6	160	2.4	129	7.3	145	9.5
4.	07040472	317	5.4	235	6.9	163	3.7	511	7.3	536	8.6	160	2.4	134	9	142	8.5
5.	07150743	317	5.4	234	15.4	167	3.7	585	9.4	536	8.6	160	2.4	141	9	140	8.5
6	07150615	317	5.4	235	15.4	167	3.7	583	9.4	536	8.6	160	2.4	134	9	141	8.5
7.	07151054	315	5.4	235	6.9	164	3.7	583	9.4	536	8.6	160	2.4	140	9	144	8.5
8.	07160229	318	5.4	233	6.9	128	3.7	583	9.4	535	8.6	160	2.4	137	9	143	8.5
9.	07160171	316	5.4	234	15.4	137	3.7	582	9.4	536	8.6	160	2.4	134	9	143	8.5
10.	07160062	317	5.4	235	6.9	140	7.9	584	9.4	536	8.6	160	2.4	134	9	143	8.5
11.	07160128	294	3.4	233	6.9	163	3.7	510	7.3	536	8.6	160	2.4	133	9	143	8.5
12.	07160129	317	5.4	232	6.9	163	3.7	582	9.4	536	8.6	160	2.4	140	9	144	9.5
13.	07170669	316	5.4	234	6.9	126	3.7	512	7.3	536	8.6	160	2.4	134	9	142	8.5
14.	07170670	317	5.4	237	6.9	145	3.7	511	7.3	536	8.6	160	2.4	135	9	142	8.5
15.	07170997	317	5.4	235	15.4	144	3.7	514	7.3	536	8.6	160	2.4	135	9	142	8.5
16	07170703	317	5.4	235	15.4	167	3.7	585	9.4	536	8.6	160	2.4	134	9	128	8.5
17.	A07180115	317	5.4	236	6.9	167	3.7	585	9.4	536	8.6	160	2.4	140	9	135	8.5
18.	A07190011	317	9.3	235	15.4	167	3.7	512	7.3	536	8.6	160	2.5	135	9	144	8.5
19.	A07190083	317	5.4	234	15.4	118	3.7	512	7.3	536	8.6	160	2.4	141	9	143	8.5
20.	A07190097	293	3.4	232	6.9	130	3.7	511	7.3	536	8.6	160	2.4	134	9	145	9.5
21.	A07190228	294	3.4	235	6.9	138	7.9	510	7.3	536	8.6	160	2.4	133	9	145	9.5
22.	P07190794	294	3.4	234	6.9	135	7.9	512	7.3	536	8.6	160	2.4	134	9	145	8.5
23.	A07190134	318	5.4	234	6.9	139	7.9	583	9.4	536	8.6	160	2.4	134	9	143	8.5
24.	A07200012	294	3.4	234	6.9	163	3.7	511	7.3	536	8.6	160	2.4	137	8	144	8.5
25.	Vaccine	317	5.4	235	15.4	166	3.7	587	9.4	535	8.6	160	2.4	139	9	-^b^	-^b^
	HGDI^c^	0.36		0.51		0.29		0.63		0.00		0.16		0.24		0.41	

a PCR fragment size based on sequencing results.

b Vaccine strain Sterne 34F2 with absence of pXO2.

c HGDI is discriminatory power based on Simpson’s formula calculated using online program ((http://insilico.ehu.es/mini_tools/discriminatory_power/index.php). PCR=Polymerase chain reaction, MLVA=Multilocus variable-number tandem repeat analysis, HGDI=Hunter Gaston diversity index

The discriminatory index of each VNTR locus was calculated based on Simpson’s formula [35,36] and estimated by the allele frequency [7,36]. The MLVA marker has a diversity of 0.00-0.63. The highest diversity was vrrC1 and the lowest was vrrC2 (Table-2). These findings were different from *B. anthracis* that was previously studied [7].

### Phylogenetic tree of East Indonesia *B. anthracis*

**Figure-3:**
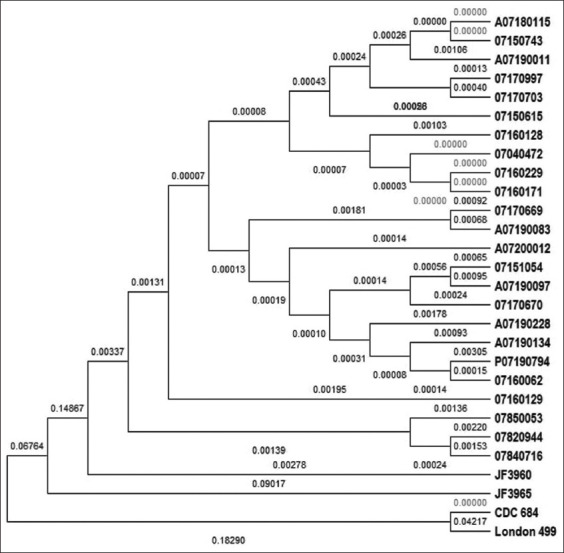
Phylogenetic tree constructed by multilocus variable-number tandem repeat analysis-8 concatenated sequences of *Bacillus anthracis* isolates circulating in East Indonesia from 1982 to 2020 using neighbor-joining p-distance method.

Based on the dendrogram generated from concatenated alignment sequences of MLVA-8, there were four subgroups of East Indonesia isolates (Figure-3). Each isolate was classified as its own genotype under lineage A, which is the most common circulating *B. anthracis* worldwide, particularly in Asia [7,9]. Four MLVA locus databases, classified as lineage A in a previous study available in GenBank, were used to construct a phylogenetic tree (JF 3960, JF 3965, London 499, and CDC 684). The old isolates (obtained in 1982, 1984, and 1985) from South Sulawesi were grouped in one subgroup (Figure-3).

All isolates included in this study were closely related, and the summary branch length was 0.565. The number of replicate trees in which the associated taxa clustered together in the bootstrap test was 1000. The evolutionary distances were computed using the p-distance with the Tamura-Nei model and are listed in the units for the number of base differences per site [38,39]. Evolutionary analyses were conducted using MEGA X. This analysis involved 28 nucleotide sequences. All positions with less than 95% site coverage were eliminated. Therefore, fewer than 5% alignment gaps, missing data, and ambiguous bases were allowed at any position (partial deletion option). A total of 1878 positions were present in the final dataset. The nucleotide frequencies are as follows: A = 39.2%, T/U=22.7%, C = 19.3%, and G = 18.8%.

## Discussion

Molecular typing of *B. anthracis* using the MLVA-8 assay revealed the genetic relationship of *B. anthracis* isolates through eight loci, consisting of six chromosomal and two plasmid markers [7,9,10,18,19]. This method has been used as a subtyping tool due to its power to distinguish between strains of many bacteria, such as *Bacillus* *cereus*, *Yersinia pestis*, *Neisseria gonorrhoeae*, and *Staphylococcus lugdunensis* [41-44]. In this study, the MLVA-8 method was used to examine the genetic characteristics and distribution of 24 field isolates. *B. anthracis* was derived from several locations/regions, hosts, and origins of samples. This is the first report of the molecular characteristics of *B. anthracis* isolates from East Indonesia that is based on the MLVA-8 method. The strains used in this study were chosen because they were well characterized and allow cross-comparison with the previous studies [7,10].

One isolate was obtained from deer at a zoo. The isolate was identified as a case of death during the quarantine period, when four deer were imported from Java to Sulawesi Island. The investigation team did not find any animal with a contact history and assumed that the quarantine facility was contaminated by fresh soil that had been used for animal bedding. The fresh soil was obtained from two districts, which are categorized as anthrax-endemic areas (Anthrax surveillance report of DIC Maros, unpublished). The first case in the Gorontalo province was an old, dried lake, which was used for cattle. The soil had been dug up to raise plants and new livestock, followed by death reports [45-49]. One investigation found that the ground ~45 years ago had been a place used for livestock burial (Anthrax Gorontalo investigation report of DIC Maros, unpublished). Both facts could be an explanation for the close relationship between the isolates from deer and the Gorontalo province and other isolates in South Sulawesi, which was the livestock production source. Although there is no direct animal-to-animal contact, it can be related to dormant spores in certain environments [48-50].

This study utilized a concatenated nucleotide of DNA sequencing results with a range of 2141-2318 bp (aligned sequences) to construct a phylogenetic tree (Table-2) rather than amino acid translation. Some mutations and deletions inside the amino acid could not be differentiated particularly due to the very low mutation rate of *B. anthracis* in the same region or a closely related outbreak [10,11,18].

All isolates showed minor differences from the DNA sequencing results for the eight marker loci (vrrA, vrrB1, vrrB2, vrrC1, vrrC2, CG3, pXO1, and pXO2). *B. anthracis* is categorized as a slow mutation rate microorganism, and the origin of all isolates is categorized by the same region [7,12]. Some deletions were identified on some molecular markers (vrrA, vrrB2, vrrC1, pXO1, and pXO2) based on fragment size. The genetic relationship from the concatenated sequences of six chromosomal and two plasmid markers showed that there was no other lineage of *B. anthracis* found in East Indonesia. This low diversity could be related to the high genetic stability of *B. anthracis*. It could be preserved for a long time under various circumstances and even under extreme environmental conditions [24,25]. The results of the TRF program for the VNTR analysis include the following information: 1) Indices of a repeat in the sequence, 2) period size, 3) number of copies aligned with the consensus pattern, 4) size of the consensus pattern, 5) percentage of matches between adjacent copies overall, 6) percentage of insertion and deletion between adjacent copies, 7) alignment score, 8) percentage composition of each of the four nucleotides (Adenine, Thymine, Guanine, Cystosine), and 9) entropy measure based on percent composition [34]. The VNTR analysis in the previous studies provided better discrimination for the polymorphism of the marker loci among strains for phylogenetic or forensic purposes for *B. anthracis* from different geographic regions [7-10].

All sequences from East Indonesia were closely related, and the overall pair distance was 0.08, with the sum of the branch length at 0.565. Most isolates (18 of 24, 75%) were from South Sulawesi, the source of cattle (and/or other livestock) in East Indonesia. The evolution of *B. anthracis*, such as the mutation or deletion process, is slower than other pathogenic bacteria [8] because it can remain dormant and be preserved for decades in the environment [3,29]. The evolution can occur during the DNA replication process but not at the spore stage [11,24]. However, the MLVA-8 assay has a limitation in terms of distinguishing isolates from closely related outbreaks [24,29]. The number of isolates for each outbreak should be increased to obtain more information and distinguish isolates based on time and representative districts [24].

## Conclusion

This study showed that MLVA-8 typing may be useful as an initial typing tool for the epidemiological investigation of identical genotypes and low genetic diversity of *B. anthracis*. No other lineage of *B. anthracis* isolates circulating in East Indonesia was found. Other molecular methods, such as extended MLVA assay (MLVA-15, MLVA-25, or MLVA-31), WGS, and canonical SNP, have been recommended for better comparative analysis and genetic study in relation to *B. anthracis* [14,15,18-22,24,25,51-54]. Further studies are needed that consider the additional loci for VNTR analysis compared to this study. However, from the fragment size of all isolates and the high percentage of homology, there may be no significant difference regarding the repeat tandem number for each marker loci.

## Authors’ Contributions

DWY: Arranged, designed, analyzed the study, and drafted the manuscript. TFD and DWY: Mapped *B. anthracis* isolates using QGIS**.** TFD, HH, and AR: *B. anthracis* collection storage, isolate, and media preparation at Bacteriology Laboratory of DIC Maros. BS and RM: Funding acquisition and administration process of the study (regarding validation of *B. anthracis* isolates data information) and supervised the laboratory experiment. BS, HS, and MS: Supervised the conceptual aspect and study design, data analysis, and revised and corrected the manuscript. All authors read and approved the final manuscript.
